# Supplementary material for: Combinatorial drug repurposing of Valproic acid and Zebularine regulates Krüppel-like factor 4 and β-catenin expression in colon cancer cells
Source: PLoS One. 2026 Jun 1;21(6):e0348215. doi: 10.1371/journal.pone.0348215 (PMC13225385; doi:10.1371/journal.pone.0348215)
Supplement: S1 File — (DOCX) [file pone.0348215.s001.docx]

**Supplementary file**

**Combinatorial drug repurposing of Valproic acid and Zebularine regulates Krüppel-like factor 4 and β-catenin expression in colon cancer cells**

Jeyalakshmi Kandhavelu^1^, Kumar Subramanian^1^, Natanya Moodley^1^, Kasim S. Abass^2^, Chandrabose Sureka^3^, Meenakshisundaram Kandhavelu^4*^, Paul Ruff ^1^ and Clement Penny^1*^

^1^Oncology Division, Department of Internal Medicine, Faculty of Health Sciences, University of the Witwatersrand, Johannesburg, South Africa

^2^College of Veterinary Medicine, Department of Physiology, Biochemistry, and Pharmacology, University of Kirkuk, 36013, Iraq

^3^Department of Basic Medical Sciences, College of Medicine, Prince Sattam Bin Abdulaziz University, Al-Kharj 11942, Saudi Arabia

^4^Molecular Signaling Group, Faculty of Medicine and Health Technology, BioMediTech, Tampere University and Tays Cancer Center, P.O. Box 553, 33101 Tampere, Finland

*Corresponding Author

[Clement.Penny@wits.ac.za](mailto:Clement.Penny@wits.ac.za) and [meenakshisundaram.kandhavelu@tuni.fi](mailto:meenakshisundaram.kandhavelu@tuni.fi)

**Figure S1.** Microscopy images of DMSO treated SW480 cells representing protein localisation for [A] KLF4 and [B] β-catenin. [A] KLF4 protein is highly condensed within nuclei, as indicated by arrows. [B] β-catenin is localised at the nuclear periphery, as revealed by the arrows. Blue fluorescence represents DAPI stained nuclei; green fluorescence represents the actin filament; red fluorescence represents the KLF and β-catenin protein; pink fluorescence represents the co-staining within the nucleus. (Original mag. 63X)

**Figure S2.** Microscopy images of DMSO treated DLD-1 cells representing protein localisation for [A] KLF4 (60x) and [B] β-catenin. [A] KLF4 protein is localised within Golgi bodies or ER at the nuclear periphery, as denoted by arrows. [B] β- catenin is found at high intensity at the cell membrane, as indicated by the arrows. Blue fluorescence represents DAPI stained nuclei; green fluorescence represents the actin filament; red fluorescence represents the KLF and β-catenin protein; pink fluorescence represents the co-staining within the nucleus. (Original mag. 63X)

**Figure S3.** Microscopy images of DMSO treated MCF-7 cells representing protein localisation for [A] KLF4 and [B] β-catenin. [A] KLF4 protein is intensely localised within the nucleus, as well as throughout the cytoplasm, as indicated by arrows. [B] β-catenin is found at high levels at the cell membrane, as denoted by the arrows. Blue fluorescence represents DAPI stained nuclei; green fluorescence represents the actin filament; red fluorescence represents the KLF and β-catenin protein; pink fluorescence represents the co-staining within the nucleus. (Original mag. 63X).

**Data used to plot figures**

|  | VPA 1.5mM | | VPA 5mM | |
| --- | --- | --- | --- | --- |
|  | KLF4 | β-Catenin | KLF4 | β-Catenin |
| MCF7 | 0.1 | -0.33 | 0.57 | 0.34 |
| SW480 | -4.78 | -0.36 | -0.31 | -5.22 |
| DLD1 | -3.53 | -1.09 | -0.17 | -3.09 |

**SD1:** Data used to plot Figure 1D: Effect of VPA on KLF4 and CTNNB1 gene expression; Logarithmic expression levels (Y axis) of KLF4 and CTNNB1 in the three cell lines, as determined by quantitative real-time PCR normalized against β-actin (ACTB).

**SD2:** Data used to plot Figure 4: Logarithmic expression levels (Y axis) of KLF4 and CTNNB1 in each cell line using quantitative real-time PCR, normalized against β-actin (ACTB).

|  | KLF4 | β-Catenin | KLF4 | β-Catenin |
| --- | --- | --- | --- | --- |
| MCF7 | 0.31 | -0.15 | 0.7 | 0.35 |
| SW480 | -3.28 | -1.03 | -0.5 | -2.3 |
| DLD1 | -0.03 | 0.002 | 0.17 | -0.29 |

**SD3:** Data used to plot Figure 6: Logarithmic expression levels (Y axis) of KLF4 and CTNNB1 in the SW480 and DLD-1 cell lines, normalized against β-actin (ACTB).

|  | KLF4 | β-Catenin | KLF4 | β-Catenin |
| --- | --- | --- | --- | --- |
| SW480 | -0.165 | -0.38 | -0.101 | -0.72 |
| DLD1 | -0.02 | -0.02 | 0.82 | -0.82 |
