# Supplementary material for: Combinatorial drug repurposing of Valproic acid and Zebularine regulates Krüppel-like factor 4 and β-catenin expression in colon cancer cells
Source: PLoS One. 2026 Jun 1;21(6):e0348215. doi: 10.1371/journal.pone.0348215 (PMC13225385; doi:10.1371/journal.pone.0348215)
Supplement: Attachment — Submitted filename: Reply to Reviewers.pdf [file pone.0348215.s003.pdf]

Manuscript Number: PONE-D-25-45571R1
Combinatorial drug repurposing of Valproic acid and Zebularine regulates Krüppel-like factor 4 and β-catenin expression in colon cancer cells

In this letter, we have replied to the comments and suggestions of the reviewers and the Editor. We thank all the reviewers for the valuable comments, and we believe that it would help us in improving the manuscript. In this letter, we addressed each question and described the changes made in accordance with all the suggestions. The changes are highlighted in **blue color** in this letter and in the manuscript. We have also replaced the schematic diagram with the table 1 and reference section has been updated accordingly.

Reply to Reviewers' comments

Reviewer #2: General Comments to the Authors:

1. The introduction is generally well structured; however, it would benefit from a more comprehensive discussion of the clinical symptoms, underlying causes, and major risk factors of colon cancer. Furthermore, a clearer linkage between these aspects and the rationale, objectives, and significance of the current study should be established to strengthen the contextual foundation of the manuscript.

Reply: Thanks for the reviewer's comments. The introduction section is revised accordingly and heighted in blue colour.

2. The manuscript should maintain consistent scientific terminology throughout. In particular, β-catenin is referred to interchangeably as CTNNB1 and β-catenin, which may cause confusion and should be standardized.

Reply: Thanks for the reviewer's comments. Since CTNNB1 is the gene that encodes the protein β-catenin, we kept the CTNNB1 terminology for discussing the gene expression while β-catenin terminology used for protein, throughout manuscript. We revised whole manuscript to avoid the confusing representation.

3. The vehicle control images corresponding to the confocal microscopy experiments should be assembled and presented together in a single figure to improve clarity, organization, and comparative interpretation of the imaging data.

Reply: Thank you for the reviewer's comments. The vehicle control images are provided in the Supplementary File. The vehicle control confocal microscopy experiments were assembled separately to allow clearer and more consistent presentation of the treated samples.

4. Vehicle control data appear to be missing from Figures 1D, 4, and 6. In the absence of these essential controls, the comparison of gene expression levels and treatment effects is scientifically inappropriate and weakens the reliability of the results.

Reply: Thanks for the reviewer's comments. In this study, the expression levels of KLF4 and β-catenin were analyzed in CRC cells using qRT-PCR, with untreated cells serving as the experimental baseline. The housekeeping gene β-actin was used as an internal control to normalize the data, accounting for variations in RNA input and reverse transcription

efficiency. Relative gene expression was calculated using the ΔΔCt method, allowing for accurate comparison of KLF4 and β-catenin levels across different treatments, including VPA, ZEB, and their combination, to assess the effects on the Wnt signaling pathway. The protocol is revised as follows "The target gene expression of KLF4 and β-catenin was quantified using qRT-PCR, normalized to the housekeeping gene β-actin, and calculated using the $2^{-\Delta\Delta Ct}$ method, with results expressed as fold changes relative to the untreated control."

5. In line 109 of the Introduction, the authors state that KLF4-overexpressing MCF-7 cells were used; however, there is no experimental evidence or methodological description confirming KLF4 overexpression. This inconsistency should be clarified and adequately supported.

Reply: Thanks for the reviewer's comments. Yes, we have stated that "The cell line, MCF-7 breast cancer cells, overexpress KLF4 have used as a positive control [22]. It is an appropriate cell line for our study because it has low expression of KLF4[23].". This statement is to justify the reason behind the selection of MCF7 cells. In addition to the previous reports, we also observed the over expression of KLF4 gene validated by qPCR and reported in Figures 1D. Fig 1D showed the one fold increase in KLF4 gene is overexpression compared to the control.

6. The tumour-suppressive role attributed to KLF4 requires stronger experimental validation. Inclusion of apoptosis-specific assays, such as flow cytometry (FACS) analysis and Western blotting of apoptotic markers, is necessary because immunofluorescence alone is insufficient to conclusively demonstrate apoptotic induction.

Reply: Thanks for the reviewer's comments. We acknowledge that assays such as FACS and Western blotting are commonly used to quantify apoptosis. However, in this study, immunofluorescence was chosen because it allows direct visualization of apoptosis-related proteins using validated, target-specific antibodies and provides important spatial and morphological information. While additional assays could further strengthen the findings, the immunofluorescence results presented here offer reliable evidence of apoptotic induction and are sufficient for the objectives of this work.

7. The reported changes in gene expression should be supported by protein-level validation. Western blotting and relevant transfection-based functional assays are required to confirm that transcriptional changes translate into meaningful biological effects.

Reply: Thanks for the reviewer's comments. We thank the reviewer for this comment. In the present study, transcriptional changes were supported by protein-level validation using immunofluorescence analysis with well-validated antibodies against KLF4 and β-catenin, allowing direct visualization of protein expression, localization, and associated morphological changes at the single-cell level. These protein-level observations were consistent with the qPCR findings and indicative of functional biological effects. While Western blotting and transfection-based assays could provide additional quantitative and mechanistic insights, the combined gene expression and immunofluorescence data presented here sufficiently demonstrate the biological relevance of the observed transcriptional changes within the scope of this study.

8. The reduction in cell proliferation does not appear to be proportionate to the reported increase in KLF4 expression. Given the modest decrease in cell viability relative to control, the claim that these effects are KLF4-dependent (as stated in lines 549–550) appears overstated and requires additional experimental justification. The use of the term "efficient" is also not adequately supported by the magnitude of the observed effects.

Reply: Thanks for the reviewer's comments. The conclusion is revised as follows "In summary, we determine that the combinatorial repurposing of VPA and ZEB may function as suboptimal anti-cancer drugs in the quest for an epigenetic treatment for colorectal cancer."

9. The proposed epigenetic effects of ZEB and VPA on histone modifications should be validated using chromatin immunoprecipitation (ChIP) assays to directly measure alterations in HDAC-associated histone marks. Reliance solely on qPCR data is insufficient to draw firm conclusions about the efficacy of the VPA+ZEB combination in colorectal cancer.

Reply: Thanks for the reviewer's comments. We thank the reviewer for the comment. VPA and ZEB are well-established epigenetic modulators (HDAC and DNMT inhibitors, respectively), and our study focused on their downstream transcriptional and phenotypic effects in colorectal cancer cells. While ChIP assays could provide direct evidence of histone modifications, the observed changes in gene expression, protein localization, and cell behavior together provide robust evidence for the efficacy of the VPA+ZEB combination. We have acknowledged this as a limitation and a potential direction for future studies.

10. There appears to be a misrepresentation of concentration values in line 384, which should be carefully corrected to ensure the accuracy and reproducibility of the experimental conditions.

Reply: Thanks for the reviewer's comments. The type is revised in section 3.6.

11. The study currently lacks direct mechanistic evidence and relies heavily on previously published literature to support its conclusions. For a manuscript that claims novelty, original experimental validation of the proposed mechanisms is required rather than extrapolation from prior reports.

Reply: Thanks for the reviewer's comments. We thank the reviewer for the comment. While previous studies have established the epigenetic roles of VPA and ZEB, our study provides original experimental evidence demonstrating their effects on KLF4 and β-catenin expression in colorectal cancer cells (SW480 and DLD-1) and control breast cancer cells (MCF-7). We validated transcriptional changes using qRT-PCR and confirmed protein localization and cellular effects via confocal microscopy, alongside functional assessments of cell viability. These data directly support the mechanistic link between VPA/ZEB treatment and modulation of key transcription factors, providing novel insight into their combinatorial anti-cancer potential in colorectal cancer.

Overall, while the study addresses an interesting and potentially relevant research question,

the manuscript requires substantial revision. Critical methodological clarifications, inclusion of appropriate controls, and stronger mechanistic and protein-level validation are essential before the conclusions can be considered reliable.

Reply: Thanks for the reviewer's comments. The conclusion in revised as follows "Our data describe the effects of valproic acid (VPA) and zebularine (ZEB), tested alone and in combination, on the transcription of KLF4 and β-catenin (CTNNB1) in colorectal cancer cell lines SW480 and DLD-1, as well as in control breast cancer cells, MCF-7. Expression of these transcription factors was analyzed by real-time RT-PCR and at the subcellular level using confocal microscopy. Additionally, the effects of VPA and ZEB on cell viability were assessed. We observed that high concentrations of VPA and ZEB increased KLF4 mRNA levels by approximately 2-fold compared with lower concentrations in early-stage SW480 cells, while the combination of the two agents at high concentrations further elevated KLF4 expression relative to the lower-dose combination. CTNNB1 was downregulated relative to KLF4. In summary, our findings suggest that the combinatorial repurposing of VPA and ZEB may serve as suboptimal anti-cancer agents, highlighting their role as epigenetic modulators in colorectal cancer."

Reviewer #3:

Reviewer #3: There are several critical inconsistencies between the mRNA data, protein-level observations, dosing rationale, and the mechanistic interpretation. These issues collectively undermine the reliability of the study's conclusions.

1. Multiple discrepancies remain between qPCR data and confocal imaging results.
• KLF4 mRNA is downregulated by VPA in both SW480 and DLD-1 cells (Fig. 1D), yet nuclear KLF4 protein staining increases markedly at 5 mM VPA (Fig. 2B).
• Low-dose ZEB decreases KLF4 mRNA (Fig. 4, pages 15–16), but KLF4 protein intensity increases at both low and high ZEB doses (Fig. 5.I.A and 5.I.B, pages 17–18).

These contradictions are neither addressed nor explained. No mechanistic rationale, methodological clarification, or acknowledgment of potential post-transcriptional or post-translational regulation is provided. This creates fundamental uncertainty about the validity of the central conclusions.

Reply: Thanks for the reviewer's valuable comment. As noted by the reviewer, yet the variation in mRNA and protein could be possible and the mechanistic insights are described in discussion section "The KLF4 mRNA downregulation and the increased nuclear KLF4 protein levels following VPA or ZEB treatment can be explained by post-transcriptional and post-translational regulatory mechanisms that are well documented in the literature. KLF4 is known to be tightly regulated at multiple levels, including mRNA stability, translational control, protein stabilization, and nuclear retention. HDAC inhibitors such as VPA can enhance protein acetylation, leading to increased protein stability and nuclear accumulation without a corresponding increase in mRNA levels. Similarly, DNA methylation inhibitors like ZEB may alter the expression of microRNAs or translational regulators that suppress KLF4 translation, resulting in enhanced protein expression despite reduced transcript levels. Previous studies have reported poor correlation between KLF4

mRNA and protein abundance due to ubiquitin-proteasome–mediated degradation, altered half-life, and context-dependent nuclear localization of KLF4 protein [49–51].”

• Under combinatorial treatment, SW480 cells show reduced KLF4 protein intensity at high dose (Fig. 7.I.B), while DLD-1 cells show substantial KLF4 upregulation at the mRNA level (Fig. 6, page 20).

Reply: Thanks for the reviewer’s valuable comment and the possible mechanistic insights are described in discussion section. “The differential effects observed under combinatorial VPA+ZEB treatment likely reflect cell line–specific regulatory mechanisms. In SW480 cells, high-dose combination treatment may enhance post-translational degradation or impair nuclear retention of KLF4 protein, leading to reduced protein intensity despite transcriptional activity. In contrast, DLD-1 cells may exhibit transcriptional de-repression of KLF4 due to cumulative epigenetic effects, highlighting stage- and context-dependent regulation of KLF4 in colorectal cancer.”

2. Although the manuscript lists the doses used (1.5 mM / 5 mM VPA and 0.1 mM / 0.3 mM ZEB), the authors provide no scientific justification for selecting these concentrations.
• No EC50/IC50 values are referenced.
• No preliminary optimization or toxicity data are presented.
• No supporting literature is cited.

Without proper justification, it is unclear whether the chosen concentrations reflect physiologic relevance, mechanistic intent, or simply reflect cytotoxicity-driven effects. This remains a major methodological limitation.

Reply: Thanks for the reviewer’s comments. We have now provided a detailed justification in introduction section showing that the chosen concentrations reflect physiological relevance, mechanistic intent, and cytotoxicity-driven effects. “In this study, we employed VPA at 1.5 and 5 mM and ZEB at 0.1 and 0.3 mM, doses selected based on prior in-vitro studies demonstrating biologically relevant epigenetic and anticancer effects. The concentrations of VPA and ZEB were selected based on prior in-vitro studies demonstrating epigenetic and anticancer activity. VPA is commonly used at millimolar concentrations in colorectal and other cancer cell lines, with reported IC_{50} values of 2–10 mM, supporting the use of 1.5–5 mM to induce HDAC inhibition without immediate nonspecific cytotoxicity [17,24,25]. Sub- to near-IC_{50} doses of VPA have also shown synergistic effects with other epigenetic agents [26]. Although fewer studies have examined zebularine in colorectal cancer models, zebularine has been shown to induce DNA demethylation and growth inhibition at 100–400 µM (0.1–0.4 mM), particularly in combination with HDAC inhibitors[27,28]. Consistently, zebularine at 50–200 µM produces dose-dependent effects in breast cancer cells, with IC_{50} values of ~88–426 µM [28]. In this study, we evaluated the demethylating and cytotoxic effects of ZEB in early-stage and late-stage colon cancer cells, explored its potential in combination with other epigenetic modifiers, and assessed its impact on the mRNA and protein expression of KLF4 and CTNNB1.”

3. The manuscript continues to assert mechanistic interactions that are not experimentally demonstrated:

• Fig. 9 (page 27) is entirely conceptual and is not supported by any functional data generated in the study.

• The Discussion (pages 23–26) repeatedly presents causal interpretations, particularly regarding Wnt signaling, apoptosis induction, and epigenetic regulation, that are not proven experimentally.

• The proposed antagonistic relationship between KLF4 and CTNNB1 is not consistently supported by the gene expression data in Fig. 6 or the single-cell protein data in Fig. 8.

• Crucially, no functional assays (e.g., KLF4 knockdown, β-catenin reporter assays, rescue experiments, chromatin studies) were performed to validate the proposed mechanism.

Reply: Thanks for the reviewer's comments. Thank you for the reviewer's comment. We have carefully re-evaluated the data and summarized the results in the table below. To avoid any potential misinterpretation, the schematic diagram has been removed and replaced with this table, which provides a clear and concise summary of the differential mRNA and protein expression of KLF4 and CTNNB1 in early- and late-stage colorectal cancer cells.

**Cell Line**	**Treatment**	**KLF4 mRNA (qPCR)**	**KLF4 Protein (IF)**	**CTNNB1 mRNA (qPCR)**	**CTNNB1 Protein (IF)**
**SW480** (Early-stage)	VPA (low dose)	↓	↔ / slight ↑	↓	↓
	VPA (high dose)	↓	↑ **(nuclear)**	↓↓	↓
	ZEB (low dose)	↓	↑	↓	↓
	ZEB (high dose)	↔ / ↓	↑	↓↓	↓↓
	VPA + ZEB (low dose)	↔	↔	↓	↓
	VPA + ZEB (high dose)	↔ / ↓	↓	↓↓	↓↓
**DLD-1** (Late-stage)	VPA (low dose)	↓	↔	↓	↓
	VPA (high dose)	↓	↑ **(nuclear)**	↓↓	↓
	ZEB (low dose)	↓	↑	↓	↓
	ZEB (high dose)	↔	↑	↓↓	↓↓
	VPA + ZEB (low dose)	↑	↔	↓	↓
	VPA + ZEB (high dose)	↑↑	↔ / modest ↑	↓↓	↓↓

Table 2: Summary of mRNA and protein expression changes following VPA and ZEB treatment- mRNA (qPCR) and protein (immunofluorescence) expression changes of KLF4 and CTNNB1 in early-stage (SW480) and late-stage (DLD-1) colorectal cancer cells following treatment with valproic acid (VPA), zebularine (ZEB), and their combination. Arrows indicate relative upregulation or downregulation compared to untreated controls.
