# Supplementary material for: Combinatorial drug repurposing of Valproic acid and Zebularine regulates Krüppel-like factor 4 and β-catenin expression in colon cancer cells
Source: PLoS One. 2026 Jun 1;21(6):e0348215. doi: 10.1371/journal.pone.0348215 (PMC13225385; doi:10.1371/journal.pone.0348215)
Supplement: Attachment — Submitted filename: Rebuttal letter with point-by-point response .docx [file pone.0348215.s002.docx]

**Response to Reviewers**

Subject: Appeal of Decision on Manuscript PONE-D-25-45571
Combinatorial drug repurposing of Valproic acid and Zebularine regulates Krüppel-like factor 4 and β-catenin expression in colon cancer cells

PLOS ONE

Dear Dr. Liu,

Thank you for your email and for sharing the reviewers’ comments regarding our manuscript entitled *"* Combinatorial drug repurposing of Valproic acid and Zebularine regulates Krüppel-like factor 4 and β-catenin expression in colon cancer cells*"*. We greatly appreciate the time and effort that went into evaluating our work. While we respect the decision, we would like to respectfully request reconsideration, as we believe some concerns may stem from misinterpretation rather than technical quality of this work.

5. Review Comments to the Author

Please use the space provided to explain your answers to the questions above. You may also include additional comments for the author, including concerns about dual publication, research ethics, or publication ethics. (Please upload your review as an attachment if it exceeds 20,000 characters)

Reviewer #1: This manuscript reported a combination drug therapy by repurposing Valproic acid and Zebularine to regulate Krüppel-like
factor 4 and β-catenin expression in colon cancer cells. However, it does not meet the publishing criteria of PLOS ONE.

1, Figure 1 shows no synergistic effects between Valproic acid and Zebularine in cell viability even though the authors claimed otherwise.

**Reply to reviewer:** Thanks for the reviewers reply. The reviewer noted that Figure 1 does not demonstrate synergy. We apologize if the presentation was unclear. We have conducted formal synergy analysis using CompuSyn Chou–Talalay report, and the results can be also presented if we have provided an opportunity. We can provide revised results with the statistical synergy data to clarify this point.

In methods, section 2.7 has been revised as follows

**“2.7. Data analysis and statistics**

All the experiments were performed with group size of n ≥ 5 independent samples. The data were represented as mean ± standard error. The experimental outcomes of VPA and ZEB were analyzed for synergism or antagonism by the Chou-Talalay combination index approach utilizing CompuSyn software. Dunnett’s multiple comparison statistical test was performed to measure the statistical significance between the tested groups; and p < 0.05 was considered as a statistically significant data.”

Consequently, Section 3.1 encompasses the interpretation of the following results:

“We also analyzed the Combination Index (CI) to determine whether the combined effect of the drugs is synergistic, additive, or antagonistic. The CI is calculated based on the dose-response curves of the individual drugs and their combinations. The obtained CI from the two combinations of VPA and ZEB (1.5+0.1 & 5+0.3) are the most suitable CI values since both are less than 1. Other hypothetical values were used to drive simulation curve in CI plot and to compute the possible CI values and to draw a comparison among the experimental and computational values. The experimental data reflects the best suitable combination of VPA+ZEB is 5+0.3mM which has 0.1 CI value. The combination of lower concentration shows complete synergy however, 5+0.3mM combination has shown better effects because in cancer therapy, synergism at high dose is more relevant to the therapy than the CI values at low dose.”

2, Figures 2,3,5 lack vehicle controls.

**Reply to reviewer:** Thanks for the reviewers reply. For Figures 2, 3, 5 Vehicle controls, DMSO was used in all experiments. Due to image size and for better representation all the controls data are given in the supplemental file.

In supplemental file following three images sets are the control images for images shown in figures 2, 3, and 5:

**Figure S.1** Microscopy images of DMSO treated SW480 cells representing protein localisation for [A] KLF4 and [B] β-catenin. [A] KLF4 protein is highly condensed within nuclei, as indicated by arrows. [B] β-catenin is localised at the nuclear periphery, as revealed by the arrows. Blue fluorescence represents DAPI stained nuclei; green fluorescence represents the actin filament; red fluorescence represents the KLF and β-catenin protein; pink fluorescence represents the co-staining within the nucleus. (Original mag. 63X).

**Figure S.2** Microscopy images of DMSO treated DLD-1 cells representing protein localisation for [A] KLF4 (60x) and [B] β-catenin. [A] KLF4 protein is localised within Golgi bodies or ER at the nuclear periphery, as denoted by arrows. [B] β- catenin is found at high intensity at the cell membrane, as indicated by the arrows. Blue fluorescence represents DAPI stained nuclei; green fluorescence represents the actin filament; red fluorescence represents the KLF and β-catenin protein; pink fluorescence represents the co-staining within the nucleus. (Original mag. 63X)

**Figure S.3** Microscopy images of DMSO treated MCF-7 cells representing protein localisation for [A] KLF4 and [B] β-catenin. [A] KLF4 protein is intensely localised within the nucleus, as well as throughout the cytoplasm, as indicated by arrows. [B] β-catenin is found at high levels at the cell membrane, as denoted by the arrows. Blue fluorescence represents DAPI stained nuclei; green fluorescence represents the actin filament; red fluorescence represents the KLF and β-catenin protein; pink fluorescence represents the co-staining within the nucleus. (Original mag. 63X).

3, Figures 1,4,6 are missing statistical analysis.

**Reply to reviewer:** Thanks for the reviewers reply. The Statistical Analysis of Figures 1, 4, 6 and all the experiments were performed with group size of n ≥ 5 independent samples. The data were represented as mean ± standard error (SEM). A Dunnett’s multiple comparison statistical test was performed to measure the statistical significance between the tested groups, and p < 0.05 was considered statistically significant.

In the previous version we noticed that the statistics analyses what we used were described in method section. Yet the P values were not shown in figures. We acknowledge that the absence of statistical annotations in the figures may have caused confusion. The Figures 1,4,6 are revised and figures legends also revise accordingly.

The revised figures and legends are given below:

**Figure 1: VPA and ZEB reduce cell viability in the SW480 and DLD-1 colon cancer cells and in the MCF-7 breast cancer cell line.** A) The cells were treated with VPA and B) ZEB individually or C) in combination, with the indicated dose of combination for 24 hrs. Carriwer control, DMSO was used a control (CT). D) Effect of VPA on KLF4 and CTNNB1 gene expression; Logarithmic expression levels (Y axis) of KLF4 and CTNNB1 in the three cell lines, as determined by quantitative real-time PCR normalized against β-actin (ACTB). Datapoints and error bars represent mean ± S.E.M (n = 6). * p < 0.05 relative to the respective DMSO control. ns, statistically non-significant.

**Figure 4: Effect of Zebularine (ZEB) on KLF4 and CTNNB1 gene expression.** Logarithmic expression levels (Y axis) of KLF4 and CTNNB1 in each cell line using quantitative real-time PCR, normalized against β-actin (ACTB). Datapoints and error bars represent mean ± S.E.M (n = 6). * p < 0.05 relative to the respective DMSO control. ns, statistically non-significant.

**Figure 6: Expression of KLF4 and CTNNB1 gene expression in colon cancer cells following combination therapy.** Logarithmic expression levels (Y axis) of KLF4 and CTNNB1 in the SW480 and DLD-1 cell lines, normalized against β-actin (ACTB). Datapoints and error bars represent mean ± S.E.M (n = 6). * p < 0.05 relative to the respective DMSO control. ns, statistically non-significant.

**Besides,** we have revised the manuscript for clarity, grammar, and readability and can provide an updated version for review.

In light of these clarifications, we believe that our manuscript meets *PLOS ONE*’s criteria for technically sound research. We therefore respectfully request that you reconsider the decision, or alternatively, permit us the opportunity to submit a revised version that fully addresses these concerns.

Thank you again for your consideration.

Sincerely,
Meenakshisundaram Kandhavelu
On behalf of all authors
