# Supplementary material for: Combinatorial drug repurposing of Valproic acid and Zebularine regulates Krüppel-like factor 4 and β-catenin expression in colon cancer cells
Source: PLoS One. 2026 Jun 1;21(6):e0348215. doi: 10.1371/journal.pone.0348215 (PMC13225385; doi:10.1371/journal.pone.0348215)
Supplement: Attachment — Submitted filename: Major Comments and Overall Assessmen re review.docx [file pone.0348215.s004.docx]

- **Major Comments and Overall Assessment- Combinatorial drug repurposing of Valproic acid and Zebularine regulates Krüppel-like factor 4 and β-catenin expression in colon cancer cells**

**The manuscript is recommended for major revision due to the following reasons:**

- The tumour-suppressive role attributed to KLF4 requires stronger experimental validation. Including apoptosis-specific assays, such as flow cytometry (FACS) analysis and Western blotting for apoptotic markers, is necessary because immunofluorescence alone is insufficient to conclusively demonstrate apoptotic induction.
- The reported changes in gene expression should be supported by protein-level validation. Western blotting and relevant transfection-based functional assays are required to confirm that transcriptional changes translate into meaningful biological effects.
- The reduction in cell proliferation does not appear to be proportionate to the reported increase in KLF4 expression. Given the modest decrease in cell viability relative to control, the claim that these effects are KLF4-dependent appears overstated and requires additional experimental justification. The use of the term “efficient” is also not adequately supported by the magnitude of the observed effects.
- The proposed epigenetic effects of ZEB and VPA on histone modifications should be validated using chromatin immunoprecipitation (ChIP) assays to directly measure alterations in HDAC-associated histone marks. Reliance solely on qPCR data is insufficient to draw firm conclusions about the efficacy of the VPA+ZEB combination in colorectal cancer.

**Concluding Statement:**

- Overall, while the study addresses an interesting and potentially relevant research question, the manuscript requires substantial revision. Stronger mechanistic and protein-level validation is essential before the conclusions can be considered reliable. Addressing these concerns will significantly improve the scientific rigour, clarity, and impact of the work. Suggested for major revision.
