# Supplementary material for: Combinatorial drug repurposing of Valproic acid and Zebularine regulates Krüppel-like factor 4 and β-catenin expression in colon cancer cells
Source: PLoS One. 2026 Jun 1;21(6):e0348215. doi: 10.1371/journal.pone.0348215 (PMC13225385; doi:10.1371/journal.pone.0348215)
Supplement: Attachment — Submitted filename: Reply to reviewers.docx [file pone.0348215.s005.docx]

**Manuscript Number: PONE-D-25-45571R1**

**Combinatorial drug repurposing of Valproic acid and Zebularine regulates Krüppel-like factor 4 and β-catenin expression in colon cancer cells**

In this letter, we respond to the comments and suggestions from the reviewers and the Editor. We thank the reviewers for their valuable feedback, which has helped improve the manuscript. We have addressed each comment and described the corresponding revisions. All changes are highlighted in blue in both this letter and the manuscript. In addition, we have also updated the references accordingly.

**Reply to reviewer 2**

*Reviewer #2: The revise manuscript does not still fully address the particular major comments mentioned in previous review which plays a significant role in proving the credibility of the study.*

**Reply to reviewer:** We thank the reviewer for this comment. We noticed that the revised sentences and paragraphs were included only in the response to reviewers in Round 2, but were not included in the manuscript. We sincerely apologize for this mistake and have now carefully added them in the appropriate sections of the manuscript. We understand that this may have given the impression that we did not fully address the previous concerns. Yet, considering the reviewer comment again we went through the major comments of reviewer 2 comments along with the reviewer 3 comments and now we have provided reply and submitted the revised version again.

**Reply to reviewer 3**

Reviewer #3: The authors have made some effort to address my previous concerns, but several critical issues remain unresolved or insufficiently addressed. My overall assessment is that the manuscript still requires major revision before it can be considered for publication.

Major Comments:

*1.1. The authors added a paragraph to the Discussion invoking post-transcriptional and post-translational regulatory mechanisms to explain the discordances between KLF4 mRNA and protein levels. While this is a plausible general explanation, it remains entirely speculative and unsupported by any data generated in this study. No mRNA stability assays, polysome profiling, proteasome inhibition experiments, or microRNA data are presented.*

**Reply to reviewer:** Thanks for the reviewer’s reply. We agree with the reviewer that many critical comments were raised; however, we partly disagree that the discussion on KLF4 mRNA and protein levels is entirely speculative as it is supported by qPCR and immunoblotting, not only at the whole-population level but also at the single-cell level. The suggested experiments on mRNA stability assays, polysome profiling, proteasome inhibition, or microRNA data are not relevant to the hypothesis of this manuscript. In review round 2, the same reviewer suggested performing “KLF4 knockdown, β-catenin reporter assays, rescue experiments, and chromatin studies.”

However, the main hypothesis of this work is that combining Valproic acid (VPA) and Zebularine (ZEB) will synergistically inhibit colorectal cancer by increasing the tumor suppressor gene *KLF4* and decreasing the oncogene *CTNNB1* (β-catenin). This is proven through in-vitro experiments on colon cancer cell lines, where combined treatment reduced cell viability and proliferation more effectively than individual drugs, showed upregulation of *KLF4* and downregulation of *CTNNB1* in qPCR and protein analyses, and induced morphological changes consistent with programmed cell death, confirming the enhanced anticancer effect of the drug combination. Since the performed cell viability assay, real-time PCR, immunofluorescence assay, imaging, and single-cell gene expression analysis provided sufficient data to test our hypothesis, we did not carry out the experiments suggested by the reviewer. We appreciate the reviewer’s suggestions on additional experiments that could advance this work; however, due to funding limitations, we plan to expand this project and implement the suggested methods wherever possible in the future.

*1.2. The response cites references 49-51 for these mechanisms, but these references do not appear in the manuscript's reference list this is a significant error that must be corrected.*

**Reply to reviewer:** Thanks for reviewer reply. The error is fixed and reference list is updated accordingly.

*1.3. Furthermore, for the combinatorial treatment discordance (SW480 vs. DLD-1), the authors offer only a qualitative, cell-line-specific rationale without experimental substantiation. The discordances between mRNA and protein data remain a fundamental unresolved issue that meaningfully weakens the study's conclusions.*

**Reply to reviewer:** Thanks for the reviewer reply. We thank the reviewer for this important comment. The observed discordance between KLF4 mRNA and protein levels is consistent across treatments and both SW480 and DLD-1, indicating a systematic pattern rather than experimental inconsistency, reported in table 2. Specifically, reduced or unchanged KLF4 mRNA alongside maintained or increased protein levels (including nuclear accumulation) suggests post-transcriptional regulation, such as altered protein stability, rather than purely transcriptional control. Importantly, this effect is gene-specific, as CTNNB1 shows consistent decreases at both mRNA and protein levels across conditions, supporting the reliability of the experimental system. The differential responses between SW480 and DLD-1 are also internally consistent and likely reflect stage-dependent biological differences rather than unexplained variability.

*2. The authors have added a paragraph to the Introduction justifying their concentration selections. This is an improvement. However, the justification cites references [24–28] that are not present in the reference list as numbered the numbering appears shifted and inconsistent with the manuscript body. This must be corrected. Additionally, the authors do not address whether VPA at 1.5–5 mM is clinically achievable in plasma or tissue — this is important context for a drug repurposing claim.*

**Reply to reviewer:** Thanks for the reviewer comment. It is revised as suggested by the reviewer. We acknowledge that the concentrations of VPA used in this study (1.5–5 mM) exceed clinically achievable plasma levels (≈0.3–0.7 mM), and therefore our findings have been interpreted as mechanistic insights rather than direct evidence of clinical efficacy. In the introduction section now we have provided the revised sentence as follows “VPA is commonly used at millimolar concentrations in colorectal and other cancer cell lines, with reported IC₅₀ values of 2–10 mM, supporting the use of 1.5–5 mM to induce HDAC inhibition without immediate nonspecific cytotoxicity [17,22,23]. However, these concentrations exceed clinically achievable plasma levels (≈0.3–0.7 mM) [24]. “

*3. The authors replaced the schematic diagram (Fig. 9) with Table 2. While this is a positive step toward transparency, the core issue remains: causal mechanistic claims about Wnt signaling, apoptosis induction, and the KLF4-β-catenin antagonism continue to be made in the Discussion without any supporting functional data. No knockdown experiments, reporter assays, rescue experiments, or ChIP data have been added. The removal of the schematic is appreciated, but it does not resolve the problem of unsupported mechanistic language. The authors should clearly reframe the Discussion using appropriately hedged, correlative language, and distinguish firmly between what was observed versus what is speculated.*

**Reply to reviewer:** Thanks for the reviewer comment. The discussion section fully revised as suggested by the reviewer. The revised sections are highlighted in blue color.

*Minor Comments:
1. The text still refers to "SYN 2 with 25 mM VPA" — this appears to be a persistent error (should be 5 mM VPA). The authors acknowledged this in their response but it must be verified as corrected in the final manuscript.*

**Reply to reviewer:** Thanks for the reviewer comment. The typo is fixed now.

*2. Line 359 refers to "HT29 and DLD-1 cells" when the study uses SW480 and DLD-1. This error remains uncorrected in the R2 manuscript and represents a serious inconsistency.*

**Reply to reviewer:** Thanks for the reviewer comment. The typo is fixed now.

*3. The abstract (line 36 of the R2 manuscript) still refers to "about one-fold" increase in DLD-1 cells in one place, while the body consistently reports a 22-fold increase. This needs reconciliation.*

**Reply to reviewer:** Thanks for the reviewer comment. The typo in online abstract is fixed now.

*4. The single-cell quantification data in Figure 8 show that ZEB at 0.1 mM appears to stimulate CTNNB1 levels in both SW480 and DLD-1 cells, which is contradictory to the proposed anti-cancer mechanism. This is acknowledged briefly in the Results but not adequately discussed.*

**Reply to reviewer: Thanks for the reviewer comment. The following description is added in the discussion section “**However, the single-cell analysis showed that the increase in CTNNB1 at 0.1 mM ZEB may be due to cellular heterogeneity, where different cells respond differently to treatment. In whole-population assays, these varied responses are averaged, which can mask opposing effects seen at the single-cell level. At low doses, ZEB may also trigger transient or adaptive responses that temporarily increase CTNNB1 in some cells before inhibition occurs at higher concentrations.”

*5. The Dunnett's test is appropriate for the comparisons described, but the manuscript continues to report some p-values inconsistently (e.g., "p > 0.0005" in section 3.4, which appears to be a typographical error and should likely be p < 0.0005).*

**Reply to reviewer:** Thanks for the reviewer comment. The typo is fixed now.

*6. The manuscript still contains grammatical errors and unclear phrasing throughout that impede scientific comprehension (e.g., "huge increase to about one-fold," "confrontational interaction," "injection of VPA and ZEB"). Professional English editing is still needed.*

**Reply to reviewer:** Thanks for the reviewer comment. The unclear phrasing has been revised throughout manuscript and grammatical errors are also fixed wherever they are needed.

*The authors have made partial progress, but the manuscript continues to make mechanistic claims unsupported by functional data, contains unresolved discordances between mRNA and protein results, has uncorrected errors (including a cell line identification error in Section 3.8 and missing/incorrect references), and retains language issues. The study's conceptual contribution exploring VPA and ZEB combination effects on KLF4 and β-catenin in CRC remains of modest interest, but the conclusions must be more carefully aligned with the actual data, and the identified errors corrected before this work can be published.*

**Reply to reviewer:** Thank you for the reviewer’s comments. We have carefully addressed all the reviewer’s points, and the manuscript has been revised accordingly. The conclusion has also been updated to better align with our main observations.
